# Utilizing mixture design response surface methodology to determine effective combinations of plant derived compounds as prostate cancer treatments

**DOI:** 10.1002/cnr2.1790

**Published:** 2023-02-11

**Authors:** Ian Geddes Berlin, Charity Conlin Jennings, Spencer Shin, Jason Kenealey

**Affiliations:** ^1^ Department of Nutrition, Dietetics, and Food Science Brigham Young University Provo Utah USA

**Keywords:** berberine, curcumin, emodin, mixture design response surface methodology (MDRSM), prostate cancer, Shikonin, silybin, traditional Chinese medicine (TCM), triptolide, wogonin

## Abstract

**Background:**

Prostate cancer (PC) is estimated to cause 13.1% of all new cancer cases in the United States in 2021. Natural bioactive compounds have drawn the interest of researchers worldwide in their efforts to find novel treatments for PC. Many of these bioactive compounds have been identified from traditional Chinese medicine (TCM) remedies often containing multiple bioactive compounds. However, in vitro studies frequently focus on the compounds in isolation.

**Aim:**

We used mixture design response surface methodology (MDRSM) to assess changes in PC cell viability after 48 h of treatment to identify the optimal mixture of all 35 three‐compound combinations of seven bioactive compounds from TCM.

**Methods and Results:**

We used berberine, wogonin, shikonin, curcumin, triptolide, emodin, and silybin to treat PC3 and LNCaP human PC cells at their IC50 concentrations that we calculated. These compounds modulate many chemotherapeutic pathways including intrinsic and extrinsic apoptosis, increasing reactive oxygen species, decreasing metastatic pathways, inhibiting cell cycle progression. We hypothesize that because these compounds bind to unique molecular targets to activate different chemotherapeutic pathways, they will act synergistically to decrease tumor cell viability. Results from MDRSM showed that two‐way combinations were more effective than three‐way or single compounds. Most notably wogonin, silybin, emodin and berberine responded well in two‐compound combinations with each other in PC3 and LNCaP cells. We then conducted cell viability tests combining two bioactive compound ratios with docetaxel (Doc) and found significant results within the LNCaP cell line. In particular, mixtures of berberine and wogonin, berberine and silybin, emodin and berberine, and emodin and silybin reduced LNCaP cell viability up to an average of 90.02%. The two‐compound combinations were significantly better than docetaxel treatment of LNCaP cells.

**Conclusion:**

Within the PC3 cells, we show that a combination of berberine, wogonin and docetaxel is just as effective as docetaxel alone. Thus, we provide new combination treatments that are highly effective in vitro for treating androgen‐dependent and androgen‐independent PC.

## 1 INTRODUCTION

Prostate cancer (PC) is the most common cancer in men in the United States. Estimates range from 1 in 10 to as high as 1 in 5 men will be diagnosed with PC. Thankfully, the overall 5‐year survival rate for PC patients is 97.8%. This statistic, however, masks the lethality of metastatic and castration‐resistant PC (mCRPC) which has a 5‐year survival rate of just 30.6%, and causes 34 000 deaths annually in the United States alone.^1^ On average, patients diagnosed with mCRPC pass away 9–13 months after diagnosis because current treatments have limited effectiveness against mCRPC.^1^, ^2^, ^3^ There is a substantial need to improve treatments against lethal metastatic, androgen‐ dependent and androgen‐ independent PC while keeping side effects in noncancerous cells to a minimum.

Treatments for PC include surgery (i.e., prostatectomy and removal of regional lymph nodes), radiotherapy, androgen deprivation therapy (ADT), and chemotherapy. Prostatectomy and radiotherapy are more frequently used for localized PC alone or in conjunction with ADT or chemotherapy. Chemotherapy, ADT, and androgen receptor (AR) inhibitors are the most common treatment options for distant PC.^4^ PC can also develop resistance to these therapies but combining therapies can improve treatment response and limit development of drug resistance.^5^ Specifically, research is being done to improve PC treatment through a combination of chemotherapy with bioactive compounds. For example, treatment of PC3 cells with docetaxel and vitamin E led to a significant decrease in the cell viability compared to docetaxel treatment alone.^6^ Researchers are also studying bioactive compounds found in traditional Chinese medicines (TCM) to determine their chemotherapeutic effects.^7^ TCM bioactive compounds are used in complex combinations, reducing the concentration of a single bioactive compound needed. The current usage of TCM bioactive compounds to treat human sickness indicates that these compounds may have low toxicity to the body. Researchers have also shown that certain TCM bioactive compounds have multiple anticancer effects. TCM bioactive compounds are a viable option to study as a treatment option in combination with chemotherapy in PC patients. Our study specifically focuses on combining TCM bioactive compounds with docetaxel as a PC treatment option. We explore combinations of seven TCM bioactive compounds, with known anticancer effects, to treat PC. Specifically, we tested three‐way combinations of TCM compounds and then tested the most effective combinations in tandem with docetaxel treatment. The compounds we chose included berberine (BB),^8^, ^9^ curcumin (Cur),^10^, ^11^, ^12^ emodin (Em),^13^, ^14^, ^15^ wogonin (Wo),^16^ shikonin (Shk),^17^, ^18^ triptolide (Ttd),^19^, ^20^ and silybin (Sy) (silibinin).^21^, ^22^, ^23^, ^24^ Each of these compounds target unique anticancer pathways (Table 1), and therefore may have synergistic effects.^8^, ^9^, ^10^, ^11^, ^12^, ^13^, ^14^, ^15^, ^16^, ^17^, ^18^, ^19^, ^20^, ^21^, ^22^, ^23^, ^24^ For example, previous studies have demonstrated that berberine increases reactive oxygen^8^ species and triptolide inhibits the androgen receptor^20^ in prostate cancer. Treating prostate cancer cells with both compounds would simultaneously target two anti‐cancer pathways which could lead to synergistic effects and lower the concentration of the drugs and reduce off target effects.

**TABLE 1:** Binding targets and anti‐cancer mechanisms of natural products

Compounds	Putative molecular target	Mechanism	References
Berberine	G‐quadruplex in the promoter region of the MYC oncogene	Decreases expression of the oncogene MYC	25
Curcumin	DYRK2	Inhibits proteosome	26
Emodin	Ser/Thr kinase CK2	Modulates cell cycle regulation	27
Wogonin	phosphatidylinositol phosphokinase	Activates apoptosis	28
Shikonin	tumor pyruvate kinase‐M2	Inhibits cancer cell glycolysis	29
Triptolide	ERCC3	Inhibits of RNA polymerase II‐mediated transcription	30
Silybin	PI3K/Akt/mTOR	Decreases cell proliferation	31

One of the most common statistical methods used to measure drug combinations is the Chou‐Talalay method because of its ability to distinguish between synergistic, antagonistic, or additive interactions.^32^ However, this method is limited because it can only test combinations of two compounds and requires them to be in constant ratios of each other. In order to test our three‐compound combinations based on the half maximal inhibitory concentration (IC50 values), we used a response surface methodology (RSM). RSMs are used to identify combination ratios that maximize a desired effect. Examples include Box–Behnken, fractional factorial, Plackett‐Burman, and mixture design response surface methodology (MDRSM).^33^ Response surface methodologies are often used in food science, engineering, and manufacturing, but used less frequently in biomedical research. We decided to use MDRSM to test our TCM bioactive compound combinations.

We used MDRSM to identify the most effective mixtures of all 35 three‐compound combinations possible from the seven TCM bioactive compounds we chose. MDRSM measures the effects of three or more compounds, requires fewer experimental runs than other response surface statistical methods, and has been used to measure three‐way chemotherapy combinations against PC in vitro.^33^ We tested the 35 three‐compound combinations in biological triplicate in three PC cell lines (PC3, LNCaP, and DU‐145).^34^, ^35^, ^36^ Both PC3 and LNCaP cell lines are derived from metastatic PC, but PC3 cells are castration‐resistant, and thus model mCRPC that is more difficult to treat.^34^, ^36^ Berberine, wogonin, emodin and silybin worked well in combination with one other compound. We further tested these four compounds in combination with docetaxel, a standard chemotherapeutic. Further, we demonstrate that the more effective combinations can reduce the dose of docetaxel needed to achieve similar outcomes.

## 2 RESULTS

### 2.1 IC50 calculations

Using the AlamarBlue cell viability assay, we determined the IC50 value for each TCM bioactive compound against PC3 cells. IC50 values are a commonly reported pharmacological property and provide a straightforward insight into the compounds' effect on cell viability. We used the IC50 value of each bioactive compound as the 100% dose in MDRSM. This allowed us to identify combinations that were more or less effective than 50% cell viability. We used IC50 values from the PC3 cells for the MDRSM. This allowed us to directly compare the responses from the cell lines at the same compound concentrations.

**FIGURE 1:**
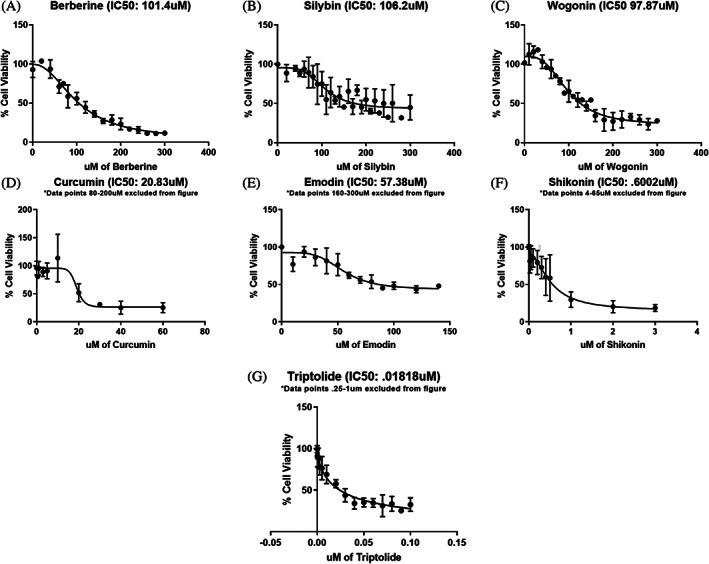
(A–G) IC50 graphs for each of the compounds used on PC3 cells. (A) berberine (BB), (B) silybin (Sy), (C) wogonin (Wo) (D) curcumin* (Cur), (E) emodin* (Em) (F) shikonin* (Shk), and (G) triptolide* (Ttd). Each point represents the average of at least triplicate biological runs. Error bars represent the standard deviation. (*Some points used to calculate the IC50 are not shown in the graph for improved clarity of the curvature for the graphical figure.)

The IC50 values we determined also gave us insight into the potency of the 7 TCM bioactive compounds. The most potent of the compounds were triptolide at 0.01818 uM (95% confidence interval (CI): 0.01094, 0.03662) and shikonin at 0.6002 uM (95% CI: 0.4191, 1.515) with IC50 concentrations in the nanomolar range (Figure 1 and Table 2). The rest of the compounds were in the micromolar range as follows: curcumin at 20.83 μM (95% CI: 17.37, 26.60), emodin at 57.38 μM (95% CI: 49.96, 65.38), wogoinin 97.87 μM (95% CI: 88.06, 110.5), berberine at 101.4 uM (95% CI: 87.37, 125.4), and silybin at 106.2 uM (95% CI: 89.13, 156.6) (Figure 1 and Table 2). Previous literature reports that the IC50s on non‐tumorigenic cells are in the high μM range for many of the compounds: shikonin (> 10 μM), wogonin (>100 μM), curcumin (192 μM), and berberine (>110 μM).^31^, ^32^, ^33^, ^34^ The other compounds in this study we could not find the IC50 values in normal cells, although our 7 TCM bioactive compounds likely have some toxicity to non‐tumorigenic cells at the IC50 concentrations calculated here, we are attempting to lower the concentration of these compounds that is necessary for treatments by identifying synergistic compounds. The rest of our work uses these IC50 values and percentages.

**TABLE 2:** Summary of IC50 values of each compound

IC50 value			
Compounds	IC50 value (μM)	95% confidence interval	R Squared
Berberine (BB)	101.4	87.37, 125.4	0.9344
Silybin (Sy)	106.2	89.13, 156.5	0.7192
Wogonin (Wo)	97.87	88.06, 110.5	0.9324
Emodin (Em)	57.38	49.96, 65.38	0.8707
Curcumin (Cur)	20.83	17.37, 26.60	0.8806
Shikonin (Shk)	0.6002	0.4191, 1.515	0.8759
Triptolide (Ttd)	0.01818	0.01094, 0.03662	0.9167

*Note*: Each compound with their abbreviation and IC50 value with its associated 95% confidence interval and R squared. The IC50 values were obtained by biological triplicate.

### 2.2 Mixture design response surface methodology (MDRSM)

**FIGURE 2:**
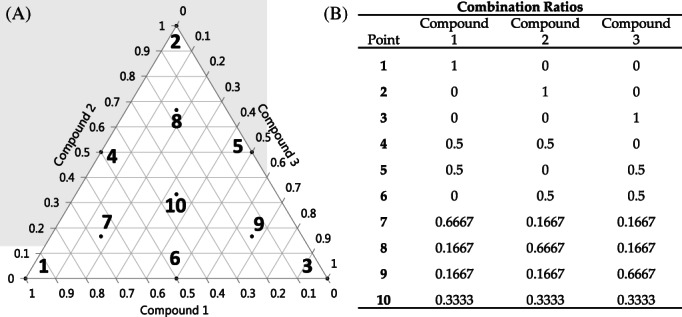
(A) Combination points shown on a response surface ternary plot. (B) MDRSM combination ratios for each of the 10 corresponding points shown in the ternary plot in A. The IC50 value was used for the proportion of 1 and fractions of the IC50 value make up the other points as shown

We tested all 35 three‐compound combinations in triplicate in LNCaP (Table 3), PC3 (Table 4), and DU‐145 (Supplemental Table 1) cells using MDRSM to predict the optimal combination by fitting the data to a response surface (Figure 2A and Supplemental Figures 1–3). This resulted in 105 unique combinations. We further tested one 3‐compound combination with MDRSM in a docetaxel‐resistant PC3 cell line (DR‐PC3) (Table 5). The ternary plots for each combination are shown in Supplemental Figures 1, 2, 3, and 4. Of the 106 combinations, 46 of them had their predicted optimal treatment to be a mixture of two compounds, and the remaining 60 combinations had an optimal treatment of just a single compound at its IC50 value. Looking at just the PC3 and LNCaP cell lines, 29 ternary plots predicted combinations while 38 ternary plots predicted single compounds as the optimal treatment. No optimal treatment predicted a combination of all three compounds in any cell line (Tables 3, 4, 5, and Supplemental Table 1).

**TABLE 3:** All 3‐way combinations in LNCaP Cells

LNCaP									
	Compounds	Proportion of compounds' IC50 concentrations	Molarity (μM) of 1st compound	Molarity (μM) of 2nd compound	Molarity (μM) of 3rd compound	Predicted % cell viability	95% confidence interval	Lack of fit Prob > *F* *	Desirability
A	Shk, BB, Wo	0, .406, .594	0	41.1684	58.13478	−2.02	10.44, −14.48	<.0001*	0.9908
B	Ttd, BB, Wo	0, .402, .598	0	40.7628	58.52626	1.22	11.21, −8.76	<.0001*	0.965
C	BB, Em, Ttd	.331, .669, 0	33.5634	38.38722	0	6.05	14.79, −2.7	0.0681	0.9086
D	Sy, BB, Wo	0, .265, .735	0	26.871	71.93445	6.09	17.83, −5.66	0.6715	0.9081
E	BB, Em, Shk	.324, .676, 0	32.8536	38.78888	0	7.12	16.71, −2.48	0.5864	0.9192
F	Cur, Em, BB	0, .749, .251	0	42.97762	25.4514	7.35	12.54, 2.16	0.5957	0.9314
G	Cur, Em, Sy	0, 1, 0	0	57.38	0	9.19	22.77, −4.4	0.6309	0.8702
H	BB, Ttd, Sy	.548, 0, .452	55.5672	0	48.0024	10.46	25.85, −4.93	0.0244*	0.9764
I	Wo, Em, Sy	0, 1, 0	0	57.38	0	11.29	22.89, −0.3	0.5019	0.8247
J	Cur, Em, Shk	0, 1, 0	0	57.38	0	11.35	27.43, −4.72	0.6057	0.9688
K	Cur, Em, Ttd	0, 1, 0	0	57.38	0	11.69	29.19, −5.8	0.3734	0.9637
L	Wo, Em, Shk	0, 1, 0	0	57.38	0	11.87	24.8, −1.06	0.6651	0.8664
M	BB, Em, Sy	.483, 0, .517	48.9762	0	54.9054	12.23	42.2, −17.74	0.9821	0.9027
N	Em, Ttd, Shk	1, 0, 0	57.38	0	0	12.29	26.39, −1.81	0.326	0.9542
O	Wo, Em, Ttd	0, 1, 0	0	57.38	0	12.74	21.65, 3.83	0.4243	0.9377
P	Em, Shk, Sy	1, 0, 0	57.38	0	0	13.78	30.43, −2.88	0.0246	0.9487
Q	BB, Cur, Sy	.493, 0, .507	49.9902	0	53.8434	14.27	39.97, −11.43	0.8623	0.8429
R	Cur, BB, Wo	0, .459, .541	0	46.5426	52.94767	14.51	30.13, −1.1	0.9735	0.7795
S	Cur, Em, Wo	0, 1, 0	0	57.38	0	14.96	32.15, −2.23	0.0483*	0.9218
T	BB, Shk, Sy	.511, 0, .489	51.8154	0	51.9318	15	32.71, −2.72	0.3582	0.9335
U	BB, Em, Wo	.251, .749, 0	25.4514	42.97762	0	16.2	29.16, 3.25	0.286	0.8814
V	Wo, Ttd, Sy	1, 0, 0	97.87	0	0	19.68	33.47, 5.9	0.3648	0.8641
W	Wo, Ttd, Shk	1, 0, 0	97.87	0	0	20.14	37.06, 3.23	0.3791	0.8833
X	Em, Ttd, Sy	.798, 0, .202	45.78924	0	21.4524	22.83	43.51, 2.14	0.9296	0.7963
Y	Cur, Wo, Shk	0, 1, 0	0	97.87	0	27.55	45.0, 10.11	0.6122	0.8902
Z	Cur, Wo, Ttd	0, 1, 0	0	97.87	0	29.98	46.87, 13.09	0.2592	0.8047
Aa	Cur, Wo, Sy	0, 1, 0	0	97.87	0	33.81	48.71, 18.91	0.495	0.7572
Bb	Cur, Shk, Sy	1, 0, 0	20.83	0	0	36.58	67.16, 5.99	0.8414	0.8474
Cc	Wo, Shk, Sy	1, 0, 0	97.87	0	0	37.19	62.85, 11.54	0.7403	0.8423
Dd	Cur, Ttd, Sy	1, 0, 0	20.83	0	0	44.01	78.31, 9.71	0.4261	0.6752
Ee	BB, Cur, Ttd	.428, .572, 0	43.3992	11.91476	0	44.11	68.03, 20.2	0.8575	0.6131
Ff	BB, Cur, Shk	.372, .628, 0	37.7208	0.3769256	0	44.22	68.37, 20.06	0.775	0.6484
Gg	Ttd, Shk, Sy	0, 0, 1	0	0	106.2	46.05	58.91, 33.2	0.295	0.7861
Hh	BB, Shk, Ttd	.915, 0, .085	92.781	0	0.00154615	58.42	69.93, 46.91	0.9637	0.7571
Ii	Cur, Shk, Ttd	1, 0, 0	20.83	0	0	62.66	78.56, 46.76	0.8755	0.8449

*Note*: Three‐compound combinations tested in LNCaP cells using mixture design response surface methodology (MDRSM). The combinations are ordered according to the predicted cell viability.

Abbreviations: BB, berberine; Cur, curcumin; Em, emodin; Shk, shikonin; Sy, silybin; Ttd, triptolide; Wo, wogonin.

* Lack of fit statistic is less than 0.05 indicating the model and experimental data do not fit well which means the other statistics predicted for this combination cannot be accepted.

**TABLE 4:** All 3‐way combinations in PC3 cells

PC3									
	Compounds	Proportion of compounds' IC50 concentrations	Molarity (μM) of 1st compound	Molarity (μM) of 2nd compound	Molarity (μM) of 3rd compound	Predicted % cell viability	95% confidence interval	Lack of fit Prob > F	Desirability
A	Shk, BB, Wo	1, 0, 0	0.6002	0	0	40.84	61.11, 20.57	0.5369	0.6479
B	Cur, BB, Wo	0, .520, .480	0	52.728	46.9776	43.22	59.59, 26.86	0.3191	0.7299
C	BB, Em, Wo	.627, 0, .373	63.5778	0	36.50551	46.31	53.83, 38.79	0.459	0.6161
D	Sy, BB, Wo	0, .448, .552	0	45.4272	54.02424	49.91	61.73, 38.09	0.924	0.844
E	Ttd, BB, Wo	0, .627, .373	0	63.5778	36.50551	51.74	61.85, 41.62	0.4736	0.7736
F	Cur, Shk, Ttd	1,0,0	20.83	0	0	51.88	61.6, 42.16	0.217	0.8164
G	Cur, Em, BB	0, .506, .494	0	29.03428	50.0916	52.86	65.12, 40.60	0.5391	0.7726
H	Cur, Wo, Shk	0, .745, .255	0	72.91315	0.153051	52.94	65.68, 40.19	0.8051	0.6897
I	BB, Cur, Shk	1,0,0	101.4	0	0	55.27	71.7, 38.85	0.4379	0.7689
J	BB, Cur, Ttd	1, 0, 0	101.4	0	0	55.48	73.41, 37.55	0.8526	0.7931
K	BB, Em, Sy	.707, 0, .293	71.6898	0	31.1166	55.88	72.98, 38.78	0.1848	0.8412
L	Cur, Wo, Sy	0, 1, 0	0	57.38	0	56.33	72.6, 40.07	0.9404	0.6609
M	Em, Shk, Sy	.514, 0, .486	29.49332	0	51.6132	57.07	65.39, 48.76	0.883	0.7438
N	Cur, Em, Sy	0, .871, .129	0	46.87946	13.6998	58.45	67.75, 49.16	0.791	0.6818
O	BB, Em, Ttd	0, 1, 0	0	57.38	0	58.56	70.02, 47.09	0.7975	0.5856
P	Em, Ttd, Sy	.597, 0, .403	34.25586	0	42.7986	58.57	62.99, 54.14	0.1998	0.8953
Q	Cur, Wo, Ttd	.484, .516, 0	10.08172	50.50092	0	59.83	68.64, 51.02	0.4764	0.7901
R	BB, Em, Shk	1, 0, 0	101.4	0	0	60.49	75.62, 45.35	0.9579	0.5886
S	BB, Ttd, Sy	.578, 0, .422	58.6092	0	44.8164	61.47	70.21, 52.73	0.363	0.758
T	BB, Shk, Sy	.627, 0, .373	63.5778	0	39.6126	61.52	72.66, 50.38	0.8598	0.8215
U	Em, Ttd, Shk	1, 0, 0	57.38	0	0	62.03	71.22, 52.85	0.9885	0.8449
V	BB, Cur, Sy	.578, 0, .422	58.6092	0	44.8164	62.06	77.19, 46.93	0.5204	0.7127
W	Cur, Em, Shk	0, 1, 0	0	57.38	0	62.56	77.89, 47.23	0.841	0.6159
X	Cur, Em, Ttd	0, 0, 1	0	0	0.01819	64.43	81.19, 47.67	0.9274	0.6097
Y	Cur, Em, Wo	0, 1, 0	0	57.38	0	64.79	80.69, 48.89	0.9428	0.6369
Z	Wo, Em, Shk	0, 0, 1	0	0	0.6002	66.13	87.0, 45.27	0.9918	0.5596
Aa	Ttd, Shk, Sy	1, 0, 0	0.01819	0	0	66.69	76.36, 57.02	0.5774	0.4248
Bb	Wo, Em, Ttd	1, 0, 0	97.87	0	0	67.33	80.81, 53.84	0.5866	0.7000
Cc	Wo, Em, Sy	1, 0, 0	57.38	0	0	69.66	82.17, 57.15	0.5587	0.7216
Dd	Cur, Ttd, Sy	1, 0, 0	20.83	0	0	69.69	86.68, 52.7	0.8853	0.6207
Ee	Wo, Ttd, Sy	.596, .404, 0	58.13478	0.00734876	0	71.59	88.37, 54.81	0.5538	0.7477
Ff	Wo, Shk, Sy	.557, .443, 0	31.96066	0.2658886	0	73.04	89.6, 56.49	0.811	0.7319
Gg	BB, Shk, Ttd	1, 0, 0	101.4	0	0	74.48	85.14, 63.82	0.7203	0.873
Hh	Wo, Ttd, Shk	0, 1, 0	0	0.01819	0	75.64	89.43, 61.86	0.9556	0.7637
Ii	Cur, Shk, Sy	1, 0, 0	20.83	0	0	84.31	94.5, 74.11	0.5211	0.7801

*Note*: Three‐compound combinations tested in PC3 cells using mixture design response surface methodology (MDRSM). The combinations are ordered according to the predicted % cell viability.

Abbreviations: BB, berberine; Cur, curcumin; Em, emodin; Shk, shikonin; Sy, silybin; Ttd, triptolide; Wo, wogonin.

**TABLE 5:** Shk, BB, Wo combination in DR‐PC3

DR‐PC3								
Compounds	Proportion of compounds' IC50 concentrations	Molarity (uM) of 1st compound	Molarity (uM) of 2nd compound	Molarity (uM) of 3rd compound	Predicted % cell viability	95% confidence interval	Lack of fit Prob > F*	Desirability
Shk, BB, Wo	0,0,1	0	0	97.87	52.17	59.84, 44.50	0.4805	0.8337

*Note*: The MDRSM combination of Shikonin (Shk), Berberine (BB) & Wogonin (Wo) used against docetaxel‐resistant PC3 cells (DR‐PC3). The results come from 4 biological runs.

Tables 3, 4, 5 and Supplemental Table 1 report the results of the MDRSM in LNCaP, PC3, DR‐PC3, and DU‐145 cells, respectively. The combinations are organized in the tables by predicted percent cell viability. The tables include the compounds used to calculate the response surface, the proportion of their IC50 value, the resulting molarity of each compound at the optimal dose, the 95% confidence interval for the predicted cell viability, and the model's lack of fit and desirability (both account for how well the data fits the model).

LNCaP cells responded most frequently to berberine, wogonin, and emodin. Berberine, wogonin, and emodin either alone or in combination contributed to 75.56% of all optimal treatments against LNCaP cells (Table 6). Combinations of berberine, wogonin and emodin also predicted high reduction of LNCaP cell viability. This is especially true in the most effective combinations even when excluding the top two optimal treatments, both of which contained berberine and wogonin, but had significant lack of fit (Table 3). The lack of fit statistic establishes that the model and data fit well. If the p‐value for the lack of fit is less than or equal to 0.05 we rejected the model due to a poor fit between the model and data. Excluding the top two optimal treatments, the top 4 optimal treatments contained combinations of berberine, wogonin, and emodin with predicted LNCaP cell viability below 8% (Table 3 (rows C–F)).

**TABLE 6:** Summary of compounds found in optimal treatments

	Berberine		Wogonin		Silybin		Curcumin		Emodin		Shikonin		Triptolide	
Cell line	BB	+	Wo	+	Sy	+	Cur	+	Em	+	Shk	+	Ttd	+
PC3	4	9	3	8	0	7	3	1	4	4	2	2	3	1
LNCaP	0	12	6	2	1	4	3	2	9	5	0	0	0	1
Total (96)*	4	21	9	10	1	11	6	3	13	9	2	2	3	2
% of total	4.17	21.88	9.38	10.42	1.04	11.46	6.25	3.13	13.54	9.38	2.08	2.08	3.13	2.08

*Note*: The compound abbreviation column represents the number of times the compound was the optimal treatment alone. The (+) column is for the number of times the compound contributed to an optimal treatment. *Of the 70 unique treatments 29 optimal treatments were combinations, each only using two compounds, the other 38 optimal treatments are a single compound leaving (29*2) + 38 = 96 total.

PC3 cells had four optimal treatments which predicted less than 50% cell viability (Table 4 (rows A–D)). Three of the optimal treatments were mixtures of berberine and wogonin (Table 4 (rows B–D)). While the most effective treatment appeared to be shikonin alone, this trend was not repeated. Berberine and wogonin consistently appeared to be part of optimal treatments contributing to 47.06% of optimal treatments against PC3 cells, whereas shikonin only contributed to 7.84% of the optimal treatments (Table 6). The predicted most effective combination of berberine and wogonin consisted of 52% BB and 48% Wo which resulted in only 43.22% predicted cell viability (Table 4 (row B)). Since berberine and wogonin were found in combination so often in the optimal treatments and reduced cells up to greater than 50%, we further tested a MDRSM combination on a docetaxel‐ resistant PC3 cell line. Previously in our lab, we grew the DR‐PC3 cell line to demonstrate chemotherapy resistant cells.^6^ We treated the DR‐PC3 cell line with a three‐way combination of shikonin, berberine, and wogonin. The optimal treatment consisted of 100% wogonin with a predicted 52.17% cell viability rate (Table 5 and Supplemental Figure 4). Although a combination of berberine and wogonin was not most effective, more research should be conducted to further determine wogonin's ability to treat chemotherapy resistant PC. From our PC3 MDRSM results, a total of 16 optimal treatments were combinations and 19 optimal treatments were single compounds; the single compounds frequently predicted less PC3 reduction than combinations (Table 4).

All 35 three‐compound combinations were run in a DU‐145 cell line as well, but the MDRSM results showed that overall single compounds were more effective than combinations (Supplemental Table 1 and Supplemental Figure 3). The top six most optimal treatments against DU‐145 cells were a single compound and the seventh most optimal treatment was barely a combination with just a small fraction of silybin combined with an overwhelming majority of curcumin (Supplemental Table 1 (row G)). The next three optimal treatments were also a single compound; overall, the top 10 optimal DU‐145 treatments essentially contained just a single compound. Curcumin was the most effective individual treatment for DU‐145 cells (Supplemental Table 1). Further research needs to be done to determine if curcumin is a viable treatment option for patients with prostate cancers similar to the isolated metastatic DU‐145 cell line. Due to this lack of mixture potency, tests on the DU‐145 line were not continued in this research. In contrast to the DU‐145 cell line, 56 out of the top 10 optimal treatments for LNCaP and PC3 cells were combinations (the top two combinations for LNCaP cells were excluded due to lack of fit).

To further show how PC3 and LNCaP cells responded to the seven compounds, we summarized the number of times each compound appeared as part of the optimal treatment from Tables 3 and 4 into Table 6. The total number of compounds in all the optimal treatments is 96 and comes from counting every time a compound is a part of the predicted optimal treatment. There were 29 two‐compound optimal treatments and 38 single compound optimal treatments which results in 96 compounds participating as part of the optimal treatments. Table 5 shows how frequently any given compound contributed to the optimal treatment indicating the potential of each compound at reducing hormone‐ responsive and non‐ responsive metastatic PC. While in combination, berberine contributed the most to the optimal treatments; berberine contributed to 21.88% of all optimal treatments. The next highest contributor, silybin, was found in the optimal combinatorial treatments 11.46% of the 96 combinations. Wogonin contributed 10.42% to all optimal treatments when in combination. Although emodin was found alone most often in the optimal treatment, it also contributed to 9.38% of all optimal treatments when in combination. Due to these four compounds contributing frequently to the optimal treatment when in combination, we continued using these compounds and combined them with docetaxel to further explore treatment options.

### 2.3 Determining optimal ratios of two compounds

**FIGURE 3:**
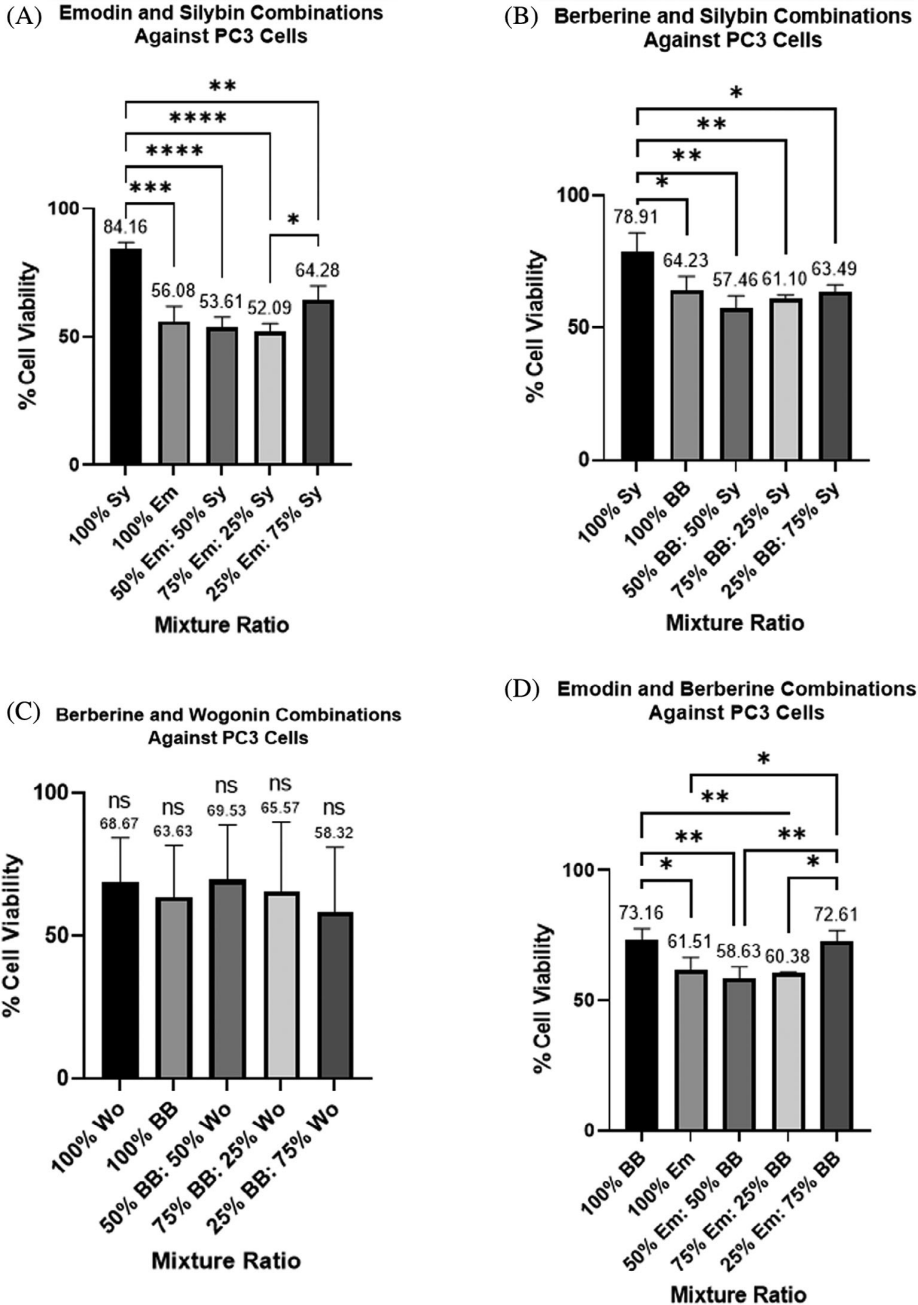
(A–D) Cell viability assays of two‐compound combinations treating PC3 cells as a percent of each compound's determined IC50 value. (A) emodin (Em) and silybin (Sy), (B) berberine (BB) and Sy, (C) BB and wogonin (Wo), (D) Em and BB. Each bar represents the average of at least triplicate biological runs. Error bars represent the standard deviation. Stars represent statistical significance between the indicated bars: *represents a *p*‐value ≤ .05, ** represents a *p*‐value ≤ .01, *** represents a *p*‐value ≤ .001, and ****represents a *p*‐value ≤ .0001. ns = not statistically different than other ratio outcomes in the same graph

**FIGURE 4:**
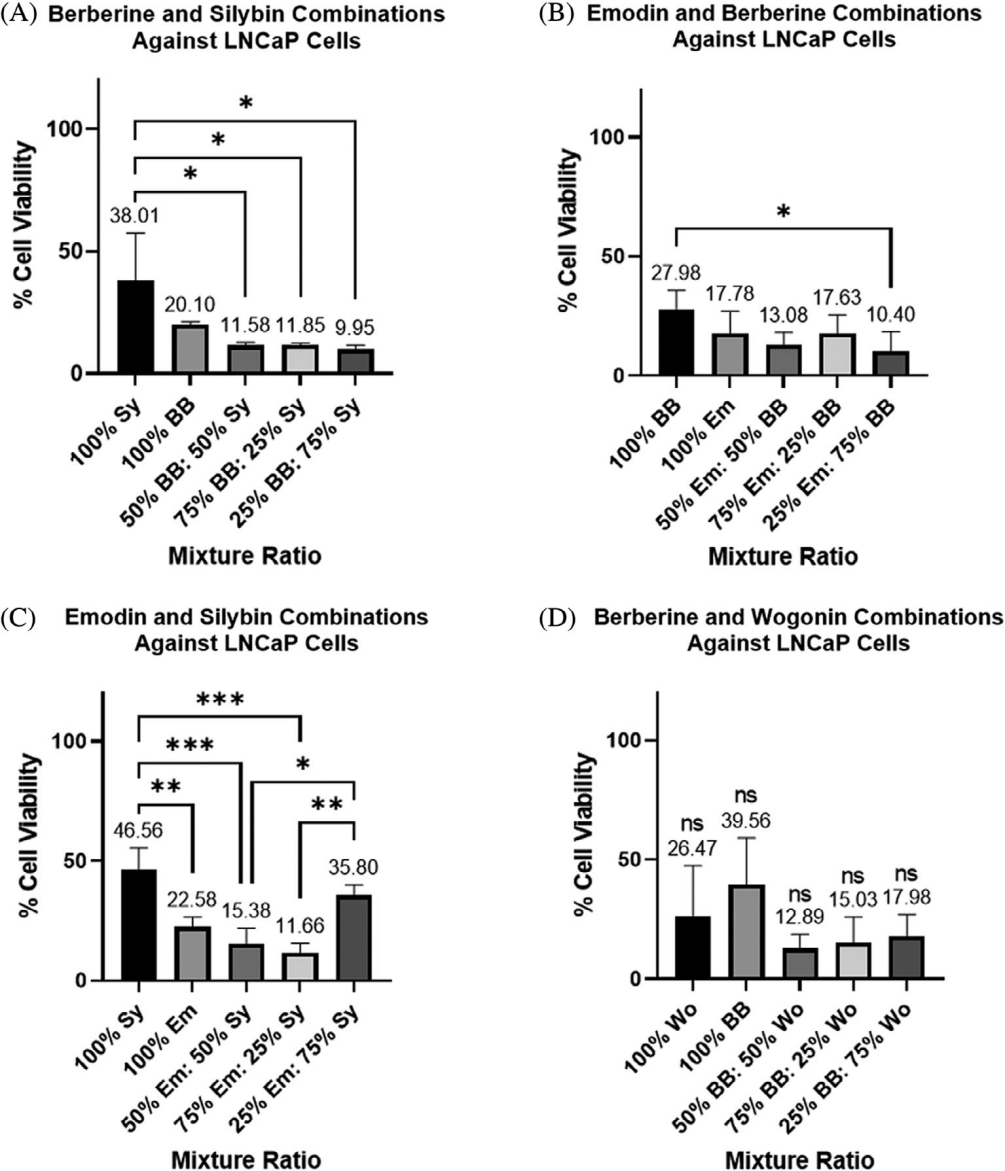
(A–D) Cell viability assays on two‐compound combinations treating LNCaP cells as a percent of each compound's determined IC50 value. (A) berberine (BB) and silybin (Sy), (B) Emodin (Em) and BB, (C) Em and Sy, (D) BB and wogonin (Wo). Each bar represents the average of at least triplicate biological runs. Error bars represent the standard deviation. Stars represent statistical significance between the indicated bars: *represents a *p*‐value ≤ .05, ** represents a *p*‐value ≤ .01, and *** represents a *p*‐value ≤ .001. ns = not statistically different than other ratio outcomes in the same graph

Based on the results above showing that some compounds were quite effective in combination, we tested combinations of berberine, silybin, wogonin, and emodin to see if they would work in tandem with docetaxel treatment. Before testing combinations of these four compounds with docetaxel, we determined optimal two‐compound combination ratios (between berberine, silybin, wogonin and emodin) through AlamarBlue cell viability tests. The two‐compound combination ratios could not be taken directly from the MDRSM ratios above because each ratio would have a third confounding variable. To determine the optimal two‐compound combination ratios, we tested (1) compound 1 at its IC50 value, (2) compound 2 at its IC50 value, (3) compound 1 at 50% of its IC50 value in tandem with compound 2 at 50% of its IC50 value, (4) compound 1 at 75% of its IC50 value in tandem with compound 2 at 25% of its IC50 value, and lastly (5) compound 1 at 25% of its IC50 value in tandem with compound 2 at 75% of its IC50 value (Figures 3 and 4).

The results for the PC3 and LNCaP cell lines showed some statistical differences between treatment ratios, but a single statistically effective ratio could not be determined as several of the mixtures decreased cell viability. Because there was not a statistically best ratio, we decided to continue tests on highly reductive ratios for each of the two‐compound combinations run. The most reductive treatment to PC3 cells consisted of a 75:25 combination of emodin: silybin which resulted in only 52.09% cell viability (Figure 3A). We see that 50% berberine and 50% silybin caused reduction of PC3 cell viability to 57.46% (Figure 3B). A 25:75 berberine:wogonin ratio resulted in PC3 cell viability of 58.32% (Figure 3C). Lastly, treatment with 50% emodin and 50% berberine caused only 58.63% of cells to remain viable (Figure 3D). Interestingly, for each of the four combinations listed above, the analyses shows that at least one of the compounds alone was not statistically different than both in combination. Since we were hoping to reduce the amount of each compound administered in an effort to reduce toxic side effects, we focused on two‐compound combinations even if not always statistically significant. Studies on the following combinations were run on PC3 cells with docetaxel treatment: 75% Em and 25% Sy, 50% BB and 50% Sy, 50% BB and 50% Wo, and 50% Em and 50% BB. These four combinations collectively will be referred to as the PC3‐4 from now on.

Similar tests were run to determine the optimal ratio of these combinations in LNCaP cells. Our tests confirmed that the % LNCaP cell viability was much more reduced by these bioactive compounds than PC3 cells. Our most reductive combination consisted of 25:75 berberine: silybin which allowed only 9.95% cell viability (Figure 4A). Surprisingly, a 25:75 ratio of emodin: berberine reduced LNCaP cells allowing only 10.40% to survive and function (Figure 4B). This is interesting when compared to the MDRSM data which shows that a 66.9: 33.1 emodin: berberine ratio was most effective within a 3 compound mixture of berberine, emodin, and triptolide (Table 3 (Row C)). This would again demonstrate that the third compound may be supressing berberine or increasing emodin effectiveness. Further tests can help shed some light on these results, but the focus of this study aims simply to find compound combinations that work in tandem with docetaxel or better than docetaxel. Similar to PC3 cells, a 75:25 emodin: silybin combination resulted in a 11.66% LNCaP cell viability (Figure 4C). These results show some potential for treating different stages of prostate cancer with the same combination ratios of emodin and silybin. Lastly, we found that a 50:50 berberine: wogonin combination was also highly reductive allowing only 12.89% cell viability although this was not statistically different than other berberine and wogonin combinations (Figure 4D). Since there was not one statistically optimal ratio for each of the four compound combinations, we continued tests on the four compound combination ratios that resulted in the lowest average % cell viability. For the LNCaP cell line, this included: 25% BB and 75% Sy, 25% Em and 75% BB, 75% Em and 25% Sy, and 50% BB and 50% Wo. These four combinations collectively will be referred to as the LNCaP‐4 from now on.

### 2.4 Determining optimal ratios of two compounds with docetaxel

**FIGURE 5:**
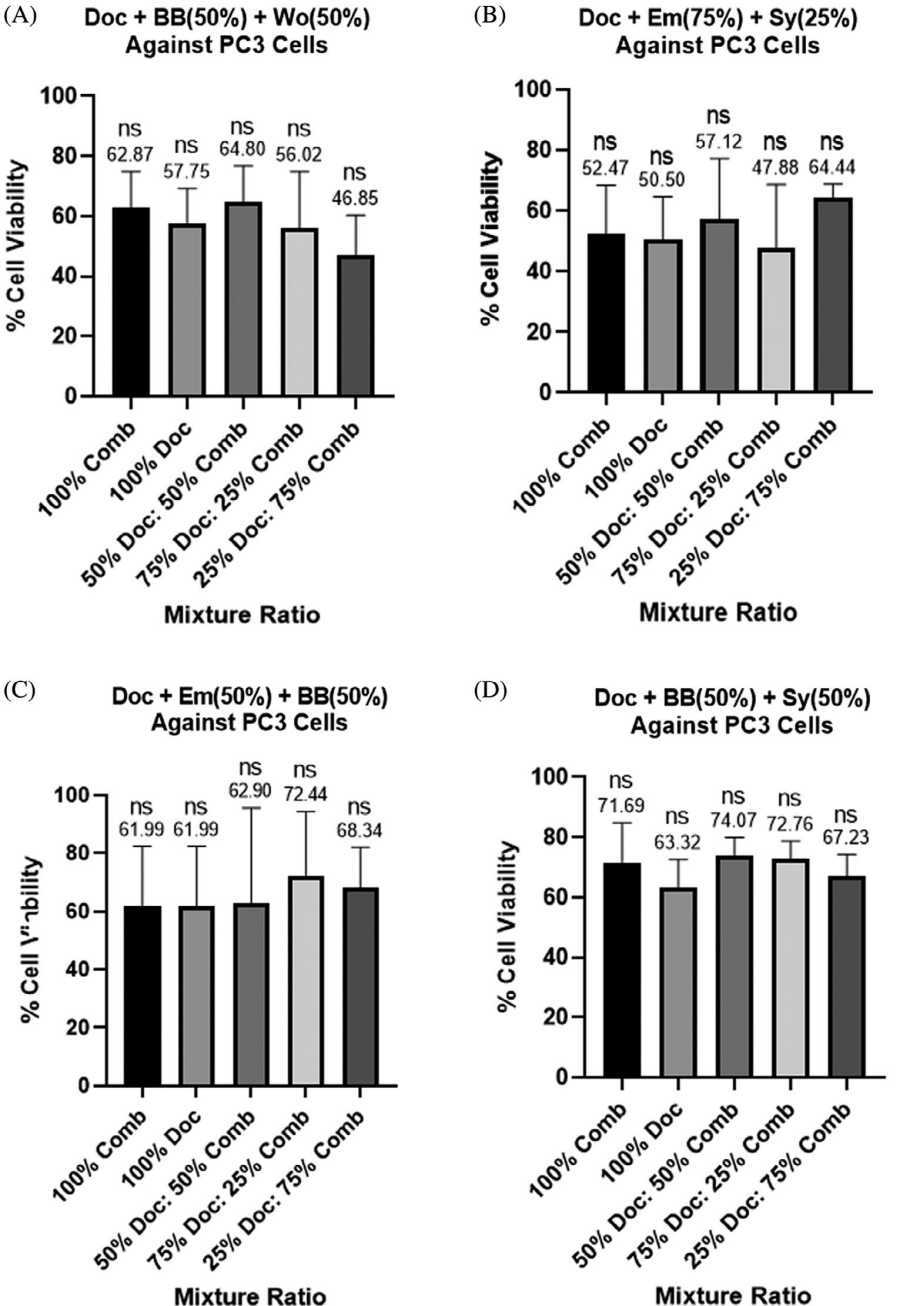
(A–D) Cell viability assays run on bioactive two‐compound combinations in tandem with docetaxel to treat PC3 cells. (A) docetaxel (Doc), berberine (BB), and wogonin (Wo), (B) Doc, emodin (Em), and silybin (Sy), (C) Doc, Em, and BB (D) Doc, BB, and Sy. In (A), 100% combination (Comb) refers to treatment with 50% BB and 50% Wo (the previously determined high averaging reductive compound combination of BB and Wo in PC3 cells). The column labeled as 100% docetaxel represents treatment with docetaxel alone at its IC50 value. The remaining bars represent mixtures of docetaxel and our previously determined high averaging reductive two‐compound combinations. In example, the 50% Doc and 50% Comb in A refers to having 50% Doc with 50% of our high averaging reductive two‐compound combination of BB and Wo consisting of 50% BB and 50% Wo. In total this would represent 50% Doc, 25% BB and 25% Wo. This applies for (B–D) as well. Each bar represents the average of at least triplicate biological runs. Error bars represent the standard deviation. ns = not statistically different than other ratio outcomes in the same graph

**FIGURE 6:**
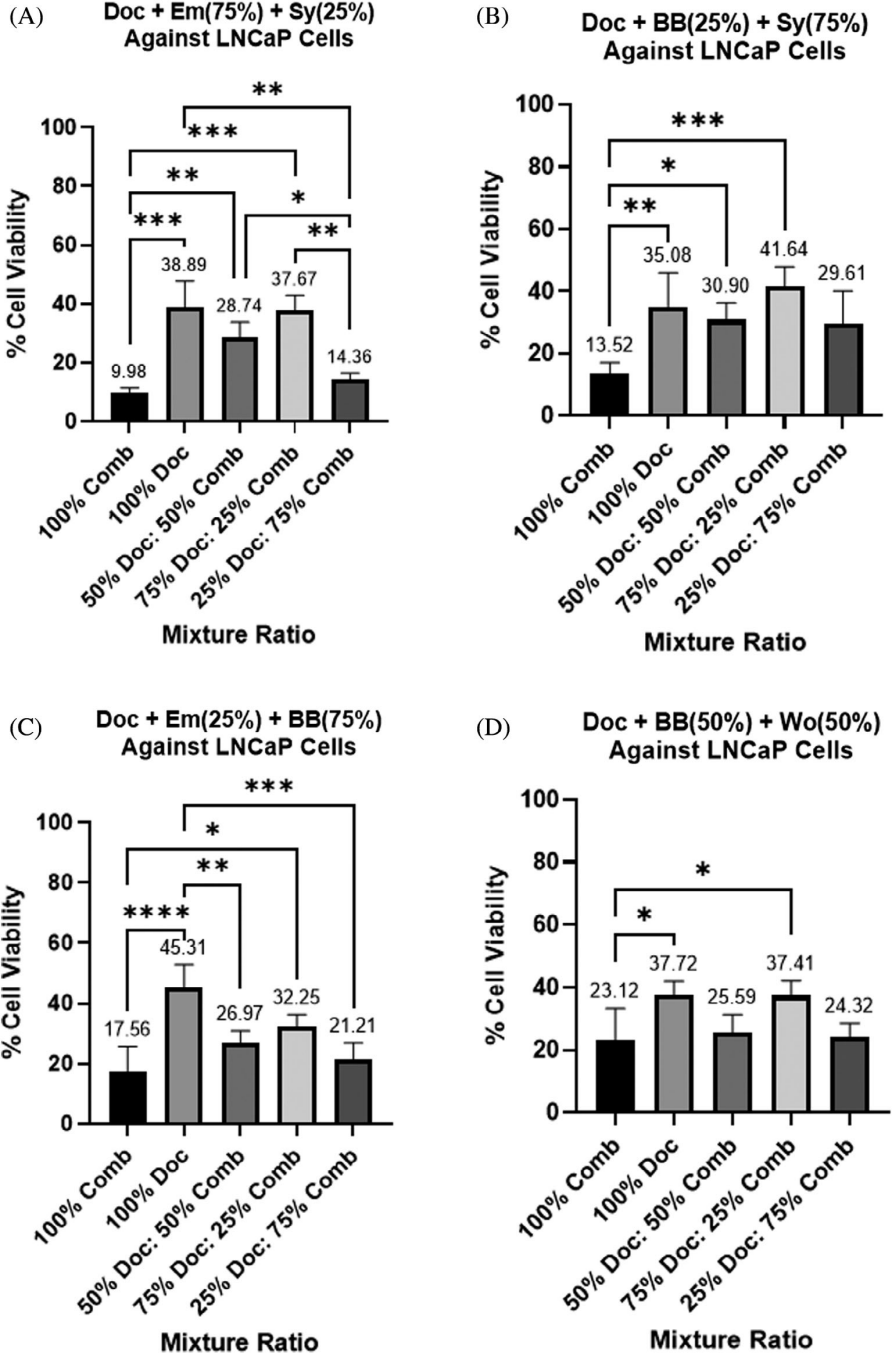
(A–D) Cell viability assays run on bioactive two‐compound combinations in tandem with docetaxel to treat LNCaP cells. (A) docetaxel (Doc), emodin (Em), and silybin (Sy), (B) Doc, berberine (BB), and Sy, (C) Doc, Em, and BB, (D) Doc, BB, and wogonin (Wo). In (A), 100% combination (Comb) refers to treatment with 75% Em and 25% Sy (the previously determined highest averaging reductive compound combination of Em and Sy in LNCaP cells). The column labeled as 100% docetaxel represents treatment with docetaxel alone at its IC50 value. The remaining bars represent mixtures of docetaxel and our highest averaging reductive two‐compound combinations. In example, the 50% Doc and 50% Comb in A refers to having 50% Doc with 50% of our highest averaging reductive two‐compound combination of Em and Sy consisting of 75% Em and 25% Sy. In total this would represent 50% Doc, 37.5% Em and 12.5% Sy. This applies for (B–D) as well. Each bar represents the average of at least triplicate biological runs. Error bars represent the standard deviation. Stars represent statistical significance between the indicated bars: * represents a *p*‐value ≤ .05, ** represents a *p*‐value ≤ .01, *** represents a *p*‐value ≤ .001, and **** represents a *p*‐value ≤ .0001

From the two‐compound combination experiments above, we treated PC3 and LNCaP cells with docetaxel and a highly reductive compound ratio (PC3‐4: 75% Em and 25% Sy, 50% BB and 50% Sy, 50% BB and 50% Wo, and 50% Em and 50% BB; LNCaP‐4: 25% BB and 75% Sy, 25% Em and 75% BB, 75% Em and 25% Sy, and 50% BB and 50% Wo). We chose to treat in combination with docetaxel because of its common use in chemotherapy and its demonstrated combination treatment effects.^4^, ^5^ Docetaxel administration was also administered as percentages of its IC50 value; docetaxel's IC50 was previously determined in our lab as 45 nM.^34^ Experiments with docetaxel were set up in the following way: (1) 1 combination of the PC3‐4, (2) docetaxel at its IC50 value, (3) docetaxel at 50% of its IC50 value in tandem with 50% of 1 of the PC3‐4, (4) docetaxel at 75% of its IC50 value in tandem with 25% of 1 of the PC3‐4, and lastly (5) docetaxel at 25% of its IC50 value in tandem with 75% of 1 of the PC3‐4 (Figures 5 and 6). This experimental set up was also run in the LNCaP cell line but with docetaxel and combinations from the LNCaP‐4.

Results from docetaxel and PC3‐4 treatments in the PC3 cell line showed no statistical difference. Although these results were not statistically different from each other, the results indicate that our two‐compound combinations in combination with docetaxel may be just as effective as docetaxel alone. A treatment combination of 25% docetaxel, 37.5% berberine and 37.5% wogonin in PC3 cells resulted in an average of 46.85% cell viability (Figure 5A). A combination of 75% docetaxel, 18.75% emodin, and 6.25% silybin reduced cells up to 52% with an average cell viability of 47.88% (Figure 5B). Interestingly, treatment with a combination of 50% emodin and 50% berberine resulted in the same % cell viability as treatment with docetaxel alone: 61.99% (Figure 5C). Lastly, docetaxel alone reduced cell viability the most when in comparison to combinations with berberine and silybin; docetaxel treatment resulted in an average PC3 cell viability of 63.32% (Figure 5D). Overall, these results show that administering just the bioactive two‐compound combinations without docetaxel can be just as reductive in PC3 cells as docetaxel administration alone. The results also provide treatment options that when in combination with docetaxel will reduce the dosage of docetaxel needed. Combining our two‐compound combinations with docetaxel may result in less negative side effects than a full dose of docetaxel. Going forward in this research, the following combinations from graphs in Figure 5 were analyzed in cell cycle experiments (a high averaging reduction of cells from each graph): 25% Doc, 37.5% BB and 37.5% Wo; 75% Em and 25% Sy; 100% Doc; and 25% Doc, 37.5% Em and 37.5% BB. This group of 4 will collectively be referred to as the PC3 ‐Doc4.

The results for the LNCaP‐ 4 with docetaxel are very promising in the LNCaP cell line. For all 4 combinations, the bioactive two‐compound combination without docetaxel was statistically more reductive than docetaxel alone (Figure 6A–D). The most reductive combination, 75:25 emodin: silybin, allowed cell viability of only 9.98%. This is about 29% less cell viability than with treatment of docetaxel alone (Figure 6A). This is really promising because previous studies have shown that silybin may target PC cells specifically. For instance, a study done by Nambiar et al showed that silybin reduces lipid accumulation in LNCaP cells preventing cancer growth without inhibiting non‐cancerous cells in the same way.^35^ The next highest averaging reduction also included silybin; a combination of 25:75 berberine: silybin resulted in just 13.52% cell viability (Figure 6B). The combination of 25:75 emodin: berberine reduced LNCaP cells leaving only 17.56% viable cells (Figure 6C). A combination of 50:50 berberine: wogonin resulted in an average LNCaP cell viability of 23.12% (Figure 6D). These results are more important to consider based on previous studies showing that wogonin significantly reduced LNCaP cells but not prostate epithelial cells.^36^ This group, which will be called the LNCaP‐ Doc4, includes: 75% Em and 25% Sy, 25% Em and 75% BB, 50% BB and 50% Wo, and 25% BB and 75% Sy.

To further determine the mechanistic actions of these compounds in combination, cell cycle analyses were run. For the LNCaP cells, these tests were run on the LNCaP‐ Doc4. However, even with high starting amounts of LNCaP cells, the cells were not able to survive the LNCaP‐ Doc4 combinations enough to get accurate cell cycle results. Cell cycle analyses were also run on the PC3 cells using the PC3‐ Doc4 (Supplemental Figure 5). The general trend showed that PC3 cells arrested in the G2/M phase if docetaxel was present in any of the PC3‐ Doc4 combinations (Supplemental Figure 5). Due to these results and previous literature, we suggest that docetaxel is having the greatest impact toward cell cycle arrest in the G2/M phase. However, the combinations we tested are reducing cell viability in some way as indicated by significant reduced cell viability even without docetaxel. We propose further research on the mechanistic effects of these compounds in combination based off of our results showing the most effective treatment ratios.

## 3 DISCUSSION

Combination therapies that are used against PC include concurrent or sequential treatment of different types of therapy such as surgery, radiation, hormone, AR inhibitors and chemotherapy.^4^ Docetaxel used earlier with ADT has been shown to extend life expectancy and improve patient outcomes,^5^ and in the 2016 STAMPEDE study, James et al. proposed that the standard of care for metastatic castration‐sensitive PC should use docetaxel along with long term ADT.^37^ In TCM, combinations may occur either by using multiple herbs or because multiple bioactive compounds are present in the same plant such as *Scutellaria baicalensis* (Chinese skullcap) which is the source of wogonin, wogoniside, baicalein, and baicalin all of which are reported to have medicinal properties.^38^ The bioactive components of TCM have been identified and their mechanisms of action is an area of increasing research interest. Each of the compounds we tested have multiple biological targets reported in the literature. Combining drugs that target different chemotherapeutic pathways can lead to synergistic effect that increase the anti‐tumorigenic effect of the combinations relative the chemotherapeutic effect compound alone.

Comparison between LNCaP and PC3 responses interestingly shows that the bioactive compounds are much more potent against LNCaP cells. Further research is needed to determine why the androgen‐dependent cell line had a larger decrease in cell viability by these bioactive compounds. Several studies demonstrate how PC3 and LNCaP cells differ in protein expression,^39^ response to microRNAs,^40^ and metabolites profile.^41^ These unique differences may explain the differential effect that that these compounds have on the two cell lines. The results from this study shed promising light on the use of these bioactive compounds with androgen‐dependent prostate cancers. Combinations of these bioactive compounds could be used after or in tandem with standard chemotherapeutic therapies to maximize treatment effectiveness and minimize deleterious side effects.

We tested the assumption that the combination of three compounds would be more effective than a single compound alone. Contrary to what we hypothesized, none of the combinations' response surface analyses predicted an optimal treatment that included all three compounds. This may have been caused by using the IC50 concentrations as the full dose treatment because MDRSM calls for fractions of the chosen full dose (i.e., 50%, 33.33%, or 16.67% of the IC50) (Figure 2), and these lower concentrations of the compound may not be biologically effective. This problem is more likely to occur with the compounds that have a stepwise shape to their IC50 curve (Figure 1). Perhaps choosing a higher drug concentration for the 100% treatments, that is points 1–3 in Figure 2A, would ensure the concentrations used at the subsequent mixture points, points 4–10 in Figure 2A, are biologically relevant for subsequent studies, as used by Asay et al. Furthermore, the MDRSM model can be improved by adding additional points to augment the simplex model and improve the fit and allow for a more exact predicted optimal treatment.^6^

To further assess the effectiveness of berberine, wogonin, emodin, and silybin, we tested highly effective two‐compound combinations in tandem with docetaxel. From our results, we see combinations of emodin and silybin, berberine and silybin, emodin and berberine, and berberine and wogonin are more effective than docetaxel alone. When used with docetaxel these combinations reduce docetaxel by as much as 75% without decreasing the chemotherapeutic efficacy. The reduction of docetaxel could reduce the nausea, diarrhea, mouth sores, and hair loss that are frequently reported as side effects of docetaxel treatment. There appears to be a characteristic in the metastatic androgen‐dependent cells that enhance their susceptibility to the two‐compound combinations. Also, this attribute seems to have changed or lessened once metastatic prostate cells become androgen‐ independent since they are less affected by the two‐ compound combinations. In an effort to see how the LNCaP cells were inhibited in their cell cycle stages, flow cytometry experiments were run. However, high enough concentrations of cells could not be maintained for flow cytometry possibly due to the high toxicity of two‐compound combinations against LNCaP cells. Further research is needed to identify the mechanisms of these discovered two‐compound combinations. Based on the mechanisms of action previously reported in the literature the two‐compound combinations likely target other pathways than the microtubules targeted by docetaxel thereby providing a multi‐pronged attack that leads to increased tumor cell death.

Due to the promising results of these two‐compound combinations, we investigated the literature explaining mechanistic actions of the four individual compounds. As small molecules, all four compounds likely bind multiple cellular targets which contributes to the broad range of effects reported in the literature. Berberine is known to modulate the inflammatory response by inhibiting NEK7‐NLRP3 interaction, IL‐1β, IL‐6, and NF‐kB, expression as well as decreasing androgen receptor expression, prostate‐specific antigen, and COX‐2 while increasing caspase‐3 and inducing apoptosis.^9^, ^39^, ^40^, ^41^ Berberine has also been noted for additional anti‐cancer effects in many in vivo studies against various cancer types and for inducing apoptosis through increasing reactive oxygen species (ROS).^8^, ^41^ Wogonin has been shown to increase p53, PUMA, Bax, and cytochrome C release from the mitochondria leading to apoptosis. Wogonin also has been reported to modulate several signal transduction pathways including inhibiting the Akt pathway to suppress tumor growth.^16^ Emodin has been reported to inhibit tumor necrosis factor‐alpha (TNF‐alpha) activation of nuclear factor kappa‐light‐chain‐enhancer of activated B cells (Nf‐kB), dysregulate mitochondrial membrane potentials, cause glutathione depletion, and generate ROS.^14^, ^15^, ^42^ Silybin has been reported to increase p21 and p27 release and cause G2 cell cycle arrest, decrease ALDH1A1, RAR alpha, Ets, and MMP9 expression.^21^, ^22^, ^23^, ^24^ It is possible that the compound combinations enhance shared mechanistic actions of the compounds or more fully inhibit proteins in the cancer cells.

In conclusion, this study puts forth two major conclusions: the use of MDRSM in cancer research and TCM compound combination treatments as effective as docetaxel which could be used with docetaxel to decrease the evolution of docetaxel resistant cells. Our results demonstrate that MDRSM is a useful statistical tool to quantify the contributions of bioactive compounds to treat PC; MDRSM requires 5–10 fewer experimental runs compared to Central Composite Design, Cross‐Correlation Function, and Box–Behnken Design.^33^ In regards to MDRSM, biological samples tend to have more variation than other areas of study, but only two out of the 106 combinations had statistically significant lack of fit which shows it is a viable statistical method for cell viability assays. The different responses we saw between cell lines, particularly the unique response of DU‐145 cells (included only in supplemental materials) compared to PC3 and LNCaP cells, highlights the need for additional studies to categorize the differences between cell‐line models from the same cancer.

Our study also indicates that compound combinations are as effective/more effective than docetaxel. In the androgen independent PC3 cell line, a combination of 37.5% BB, 37.5% Wo and 25% Doc reduced cell viability at an average of 53%. This treatment combination was just as effective as docetaxel treatment alone. After a patient's PC becomes androgen independent and progresses on or after a 2nd generation AR inhibitor, the patient is then usually treated with chemotherapy such as docetaxel. We propose further research on this combination of docetaxel, berberine and wogonin as a treatment option for androgen‐ independent patients before they receive full dosages of chemotherapies. In the androgen‐ dependent LNCaP cell line, all 4 two‐compound combinations significantly decreased cell viability in comparison to docetaxel. Specifically, 75% Em and 25% Sy reduced LNCaP cells almost 30% more than docetaxel treatment (Figure 6A). These 42‐compound combinations should be tested further as potential treatment for PC patients while they are using androgen deprivation therapies and before they become androgen‐ independent. Lastly, our studies show that single compound wogonin has significant toxicities to docetaxel‐resistant PC3 cells. These docetaxel resistant cells treated with wogonin had an average of only 52.17% cell viability (Table 5). This is a promising potential treatment as chemotherapy is typically the last line for patients besides changing to different chemotherapies or different AR inhibitors. Further studies on our identified two‐compound combinations and wogonin in docetaxel resistant PC models are needed to determine the efficacy of these treatments in organisms.

## 4 MATERIALS AND METHODS

### 4.1 Cell lines

We obtained human prostate cancer PC3 (CRL‐1435), DU‐145 (HTB‐81), and LNCaP (CRL‐1740) cells from ATCC (Rockville, MD, USA). The PC3 cells were incubated in F‐12 K, 1X (Ham's F‐12 K Nutrient Mixture, Kaighn's Mod.) with L‐glutamine (10‐025‐CV) purchased from Corning Incorporated (Oneonta, NY, USA). The DU‐145 cells were incubated in Eagle's Minimum Essential Medium (30–2003) purchased from ATCC. The LNCaP cells were grown in RPMI‐1640 (30–2001) media purchased from ATCC. For each cell line, we added 10% fetal bovine serum (Corning, 35‐010‐CV) and 1% antibiotic (Hyclone, SV30010) and kept them in a 37°C humidified 5% CO2 incubator from passages 4–30. Docetaxel‐Resistant Clones were previously prepared in the lab as reported by Asay 2020.^6^

### 4.2 Compounds

We purchased the bioactive compounds from Cayman Chemical (Ann Arbor, Michigan, USA). Their catalog numbers are listed after each compound: berberine (633‐65‐8), curcumin (458‐37‐7), emodin (518‐82‐1), triptolide (38748–32‐2), wogonin (632‐85‐9), shikonin (517‐89‐5), and silybin (22888‐70‐6)*. The dry powder compounds were dissolved in DMSO (Millipore, 67‐68‐5) to make 100 mM stock solutions and aliquoted out into microcentrifuge tubes to minimize freeze thaw cycling.

*Cayman lists product number 10006211 as silybin, however from PubChem it appears the compound may have been a stereoisomer of silybin called silibinin.^43^, ^44^

### 4.3 Cell viability

To assess cell viability, we used the AlamarBlue cell viability assay after PC3 and LNCaP cells were grown to confluence, trypsinized with 0.25% Trypsin‐EDTA (VWR, 02‐0154‐0100), and plated in Greiner bio‐one Cellstar 96 well plates (Millipore Sigma, M0812) at 5000–10000 cells per well with 100 μl of medium. We allowed each cell line to adhere to the bottom of the 96‐well plate for a 24‐h period before treatment. Each 96‐well plate had a DMSO vehicle control set to 100% viability with which the other treatments were normalized to. After 48 h of incubation, 10 μL of AlamarBlue (BioRad, BUF012A) was added to each well containing cells and placed in the incubator for an additional 5–6 h. A time period of 48 h was chosen based off of previous literature which determined some of the compounds had greater cytotoxic effects on cancer cells with longer treatment times.^9^, ^12^, ^13^ Researchers also found that compounds such as berberine had a smaller effect on non‐malignant cells at shorter time points.^8^ Thus, we decided to proceed with the medium time period commonly used in PC viability assays, 48 h, and only ran this time point due to the scope of the experiment (315+ assays). Cell viability was analyzed via relative fluorescence as read by BMG LABTECH FLOUstar OPTIMA at 544 nm excitation and 612 nm emission, BioTek (Winooski, VT, USA) using fluorescence measurement at 540/35 nm excitation filter and a 590/20 nm emission filter, Victor Nivo (PerkinElmer Inc. Waltham, MA, USA) with a 530/30 nm excitation filter and a 595/10 nm emission filter, and Spectra Max iD3 with a 540 nm excitation filter and a 590 nm emission filter (Molecular Devices San Jose, CA, USA).

### 4.4 IC50 value calculation and statistical analysis

We used GraphPad Prism 8 (Graphpad Software, San Diego, CA, USA) to calculate IC50 values for each of the 7 compounds used using gradation treatment and following the cell viability method stated in section 4.3. Using concentrations above and below the IC50 concentration, a variable slope non‐linear regression model was fit to the experimental results (*r* ^2^ > 0.95). The concentration which resulted in a 50% reduction of cell viability was set as the IC50 for each compound based on at least 3 biological runs. We also used GraphPad Prism 8 to run statistical analyses on Figures 3, 4, 5, 6. The data was first tested for outliers using the ROUT method with a *Q* = 10%. Then the data was analyzed to determine statistical significance using the Ordinary one‐way ANOVA, Tukey's multiple comparisons test (Figures 3, 4, 5, 6).

### 4.5 Mixture design response surface methodology

MDRSM builds a response surface with an axis for each compound tested. Because we used three compounds, MDRSM built a ternary plot (Figure 2A) that had 10 mixtures of the three compounds (Figure 2B). Point one is 100% of compound 1 just as point two is 100% of compound 2 and point three is 100% of compound 3. We used the IC50 value of each compound as the 100% value, or full dose treatment. The points within the ternary plot are proportions of the full dose of each compound used at the vertices; for example, point four is 50% of compounds 1 and 2, point seven is 66% of compound 1 and 16% of compounds 2 and 3, and point 10 is 33% of each compound. Again, percentages are based off each compound's IC50 value. MDRSM uses the experimental data from these 10 points to build a statistical model that predicts the optimal combination of the chosen compounds.

We analyzed the compound combinations using JMP Pro15 software (SAS Institute, Cary, NC, USA). ABCD mixture design was used for factor analysis and generation of the ternary plots. The methods followed Oblad et al methods.^34^ We used the same simplex lattice augmented with four additional points resulting in the 10 experimental points; by using the least‐squares method, coefficients were estimated for use in the quadratic mixture model. Each 96‐well plate had the 10 mixture treatments and was normalized to a DMSO vehicle control which was set at 100% viability.

### 4.6 Flow cytometry

Cells were plated on 6 well plates and allowed to adhere for a 24‐hour period. Cells were then treated. Each 6‐well plate had a DMSO vehicle control that was set to 100% viability with which the other treatments were normalized to; each plate also had a cell‐free control. After 48 h, cells were trypsinized and received 500 μl of 70% ethanol dropwise while being vortexed. Cells were then refrigerated for 3–4 h at 4°C. Next cells were centrifuged and washed with 1× PBS (Fisher Bioreagents, BP399‐1). After adding 200 μl of Propidium iodide, made at 50 μg/ml in 1× PBS (Caymen Chem, 14 289), and 1.3 ml of 1× PBS, cells were vortexed. Cells were then run and analyzed by the Accuri C6+ Flow Cytometer (BD Biosciences, Mississauga, ON, Canada).

## AUTHOR CONTRIBUTIONS

**Ian Berlin:** Conceptualization (equal); data curation (equal); formal analysis (equal); investigation (equal); methodology (equal); visualization (equal); writing – original draft (equal); writing – review and editing (equal). **Charity Jenning:** Conceptualization (equal); data curation (equal); formal analysis (equal); investigation (equal); methodology (equal); visualization (equal); writing – original draft (equal); writing – review and editing (equal). **Spencer Shin:** Investigation (equal); writing – review and editing (supporting). **Jason Kenealey:** Conceptualization (equal); funding acquisition (equal); methodology (equal); project administration (equal); supervision (equal); visualization (equal); writing – original draft (equal); writing – review and editing (equal).

## FUNDING INFORMATION

This research has been supported by a generous donation from Bryant Adams, the Simmons Center for Cancer Research, and CURA grants.

## CONFLICT OF INTEREST

The authors have stated explicitly that there are no conflicts of interest in connection with this article.

## ETHICS STATEMENT

None.

## Supporting information


**Appendix S1:** Supporting information.

## Data Availability

The data that support the findings of this study are available from the corresponding author upon reasonable request.
