# Supplementary material for: Utilizing mixture design response surface methodology to determine effective combinations of plant derived compounds as prostate cancer treatments
Source: Cancer Rep (Hoboken). 2023 Feb 11;6(4):e1790. doi: 10.1002/cnr2.1790 (PMC10075293; doi:10.1002/cnr2.1790)
Supplement: Supplementary file 1 — Appendix S1: Supporting information. [file CNR2-6-e1790-s001.docx]

# Appendix A

Supplemental Figures

Supplemental Table 1. All 3-way combinations in DU-145 cells. Three-compound combinations tested in DU-145 cells using mixture design response surface methodology (MDRSM). The combinations are ordered according to the % cell viability. Cur, curcumin; Shk, shikonin; BB, berberine; Wo, wogonin; Sy, silybin; Em, emodin; Ttd, triptolide

Supplemental Figure 1. All ternary plots of LNCaP cells arranged from the combination that caused the greatest predicted decrease in cell viability to the least. Each graph uses its own color key that is optimized to show different levels of significance. Each point within the same shade of red has no statistical difference, although for each ternary graph a single ideal point is predicted. Combinations A-Ii corresponding data is presented in Table 2.

Supplemental Figure 2. All ternary plots of PC3 cells arranged from the combination that caused the greatest predicted decrease in cell viability to the least. Each graph uses its own color key that is optimized to show difference levels significance. Each point within the same shade of red has no statistical difference, although for each ternary graph a single ideal point is predicted. Combinations A-Ii corresponding data is presented in Table 3.

Supplemental Figure 3. All ternary plots of DU-145 cells arranged from the combination that caused the greatest predicted decrease in cell viability to the least. Each graph uses its own color key that is optimized to show different levels of significance. Each point within the same shade of red has no statistical difference, although for each ternary graph a single ideal point is predicted. Combinations A-Ii corresponding data is presented in Supplemental Table 1.

Supplemental Figure 4. Ternary plot of the combination from shikonin, berberine and wogonin. The associated statistics are in Table 4.

Supplemental Figure 5. Cell cycle analyses of PC3 cells after treatments. Two-Way ANOVA test to determine statistical difference of treatments from controls. * Signifies statistically different than DMSO control. # Represents statistically different than docetaxel alone.

Supplemental Table 1:

| DU-145 | | | | | | | | | |
| --- | --- | --- | --- | --- | --- | --- | --- | --- | --- |
|  | Compounds | Proportion of compounds' IC50 concentrations | Molarity (uM) of 1st compound | Molarity (uM) of 2nd compound | Molarity (uM) of 3rd compound | Predicted decrease in % cell viability | 95% confidence interval | Lack of fit Prob>F | Desirability |
| A | Cur, Shk, Ttd | 1, 0, 0 | 20.83 | 0 | 0 | 22.51 | 40.6, 4.47 | 0.0712 | 0.8463 |
| B | BB, Cur, Ttd | 0, 1, 0 | 0 | 20.83 | 0 | 28.67 | 43.64, 13.71 | 0.1536 | 0.8765 |
| C | Wo, Em, Sy | 1, 0, 0 | 97.87 | 0 | 0 | 28.78 | 43.03, 14.54 | 0.8862 | 0.7986 |
| D | Cur, Wo, Sy | 1, 0, 0 | 20.83 | 0 | 0 | 29.96 | 47.85, 12.06 | 0.984 | 0.7654 |
| E | Wo, Em, Shk | 1, 0, 0 | 97.87 | 0 | 0 | 30.45 | 51.08, 9.82 | 0.9789 | 0.7323 |
| F | Cur, Em, Ttd | 1, 0, 0 | 20.83 | 0 | 0 | 32.76 | 53.6, 11.93 | 0.9579 | 0.8264 |
| G | Cur, Ttd, Sy | .958, 0, .042 | 19.95514 | 0 | 4.4604 | 33.12 | 48.26, 17.99 | 0.313 | 0.8541 |
| H | Cur, Wo, Ttd | 1, 0, 0 | 20.83 | 0 | 0 | 34.01 | 49.67, 18.35 | 0.5925 | 0.8111 |
| I | Cur, Em, BB | 0, 1, 0 | 0 | 57.38 | 0 | 34.39 | 59.08, 9.71 | 0.9099 | 0.6101 |
| J | BB, Em, Wo | 0, 1, 0 | 0 | 57.38 | 0 | 35.97 | 56.07, 15.87 | 0.1972 | 0.6653 |
| K | Sy, BB, Wo | 0, .474, .526 | 0 | 48.0636 | 51.47962 | 36.36 | 57.52, 15.21 | 0.9685 | 0.8224 |
| L | Wo, Em, Ttd | 0, 1, 0 | 0 | 57.38 | 0 | 36.67 | 52.52, 20.81 | 0.9379 | 0.7494 |
| M | Cur, Em, Wo | 0, 1, 0 | 0 | 57.38 | 0 | 36.85 | 66.29, 7.41 | 0.9994 | 0.6553 |
| N | Cur, BB, Wo | 0, .445, .555 | 0 | 45.123 | 54.31785 | 37.04 | 49.21, 24.87 | 0.0833 | 0.8677 |
| O | Cur, Wo, Shk | 1, 0, 0 | 20.83 | 0 | 0 | 37.52 | 51.0, 24.04 | 0.6593 | 0.76809 |
| P | Em, Shk, Sy | 1, 0, 0 | 57.38 | 0 | 0 | 38.91 | 54.21, 23.6 | 0.6701 | 0.7512 |
| Q | Em, Ttd, Sy | 1, 0, 0 | 57.38 | 0 | 0 | 42.7 | 48.45, 36.94 | 0.2144 | 0.805 |
| R | Wo, Shk, Sy | 1, 0, 0 | 97.87 | 0 | 0 | 44.9 | 58.71, 31.1 | 0.5601 | 0.8204 |
| S | BB, Cur, Sy | 0, .824, .176 | 0 | 17.16392 | 18.6912 | 44.92 | 58.96, 30.89 | 0.9696 | 0.678 |
| T | Ttd, BB, Wo | 0, .482, .518 | 0 | 48.8748 | 50.69666 | 45.57 | 57.92, 33.23 | 0.5511 | 0.8916 |
| U | Cur, Em, Sy | 0, 1, 0 | 0 | 57.38 | 0 | 45.74 | 60.88, 30.6 | 0.7412 | 0.8113 |
| V | Em, Ttd, Shk | 1, 0, 0 | 57.38 | 0 | 0 | 46.77 | 66.07, 27.46 | 0.9745 | 0.748 |
| W | Shk, BB, Wo | 0, .500, .500 | 0 | 50.7 | 48.935 | 46.88 | 58.18, 35.57 | 0.3417 | 0.8864 |
| X | Cur, Shk, Sy | 1, 0, 0 | 20.83 | 0 | 0 | 47.55 | 66.02, 29.08 | 0.2852 | 0.7916 |
| Y | BB, Em, Shk | .194, .806, 0 | 19.6716 | 0.4837612 | 0 | 47.89 | 61.25, 34.52 | 0.7572 | 0.8539 |
| Z | BB, Cur, Shk | 0, 1, 0 | 0 | 20.83 | 0 | 50.31 | 71.02, 29.6 | 0.9064 | 0.6536 |
| Aa | Wo, Ttd, Sy | .679, .321, 0 | 66.45373 | 0.00583899 | 0 | 51.3 | 59.9, 42.69 | 0.8279 | 0.7509 |
| Bb | BB, Ttd, Sy | .609, 0, .391 | 61.7526 | 0 | 41.5242 | 51.45 | 62.8, 40.11 | 0.5255 | 0.6829 |
| Cc | BB, Shk, Sy | .600, 0, .400 | 60.84 | 0 | 42.48 | 52.97 | 66.89, 39.04 | 0.923 | 0.7017 |
| Dd | Cur, Em, Shk | 0, 1, 0 | 0 | 57.38 | 0 | 55.51 | 71.8, 39.21 | 0.928 | 0.636 |
| Ee | BB, Em, Ttd | .254, .746, 0 | 25.7556 | 42.80548 | 0 | 55.56 | 69.87, 41.24 | 0.7285 | 0.7047 |
| Ff | Wo, Ttd, Shk | .768, .232, 0 | 75.16416 | 0.00422008 | 0 | 56.33 | 64.59, 48.06 | 0.985 | 0.7827 |
| Gg | BB, Em, Sy | 0, 1, 0 | 0 | 57.38 | 0 | 61.07 | 71.98, 50.16 | 0.6622 | 0.7248 |
| Hh | Ttd, Shk, Sy | .586, 0, .414 | 0.01065934 | 0 | 43.9668 | 69.53 | 82.67, 56.4 | 0.437 | 0.6644 |
| Ii | BB, Shk, Ttd | .684, 0, .316 | 69.3576 | 0 | 0.00574804 | 80.64 | 91.12, 70.16 | 0.8222 | 0.581 |

Three-compound combinations tested in DU-145 cells using mixture design response surface methodology (MDRSM). The combinations are ordered according to the predicted cell viability. Cur, curcumin; Shk, shikonin; BB, berberine; Wo, wogonin; Sy, silybin; Em, emodin; Ttd, triptolide

Supplemental Figure 1


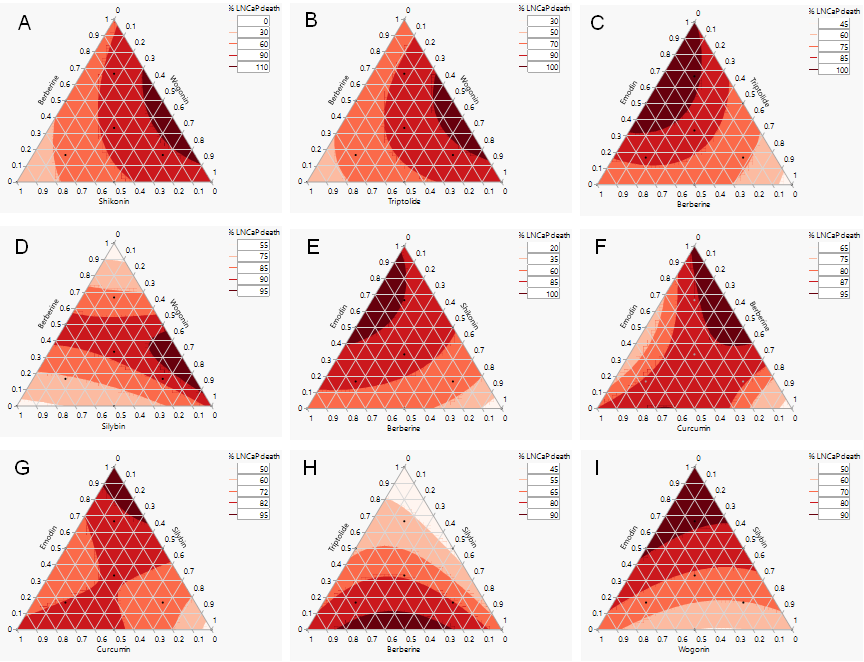


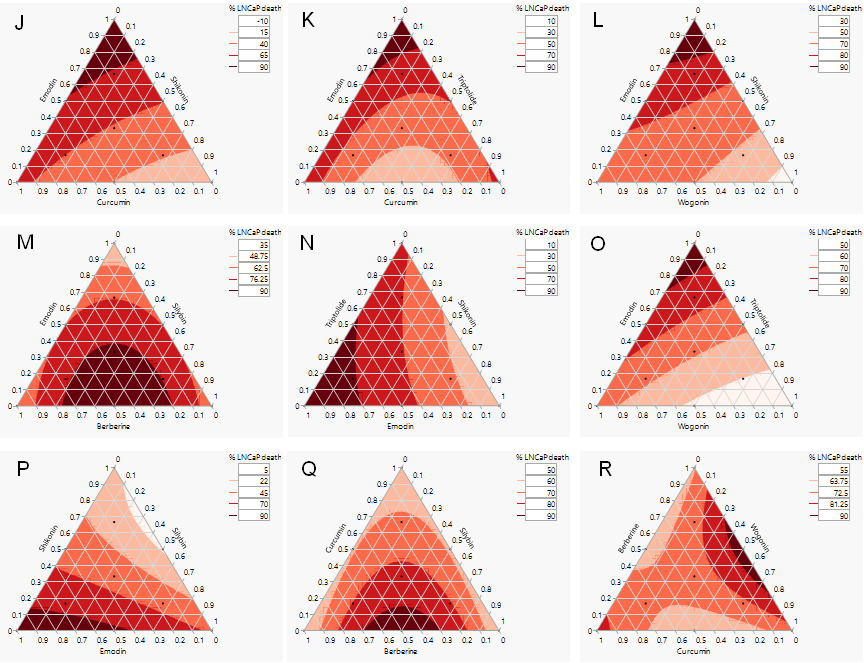


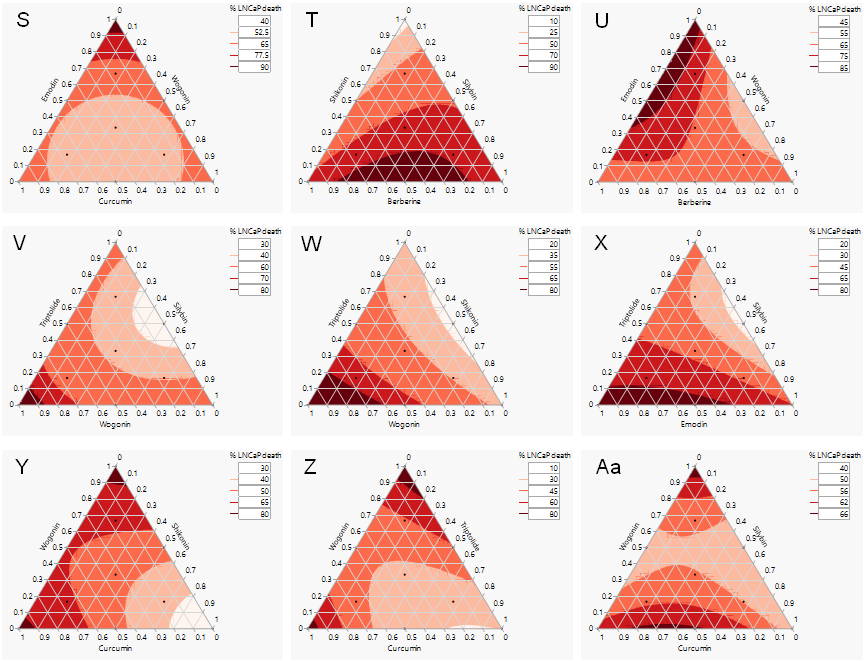


Supplemental Figure 2


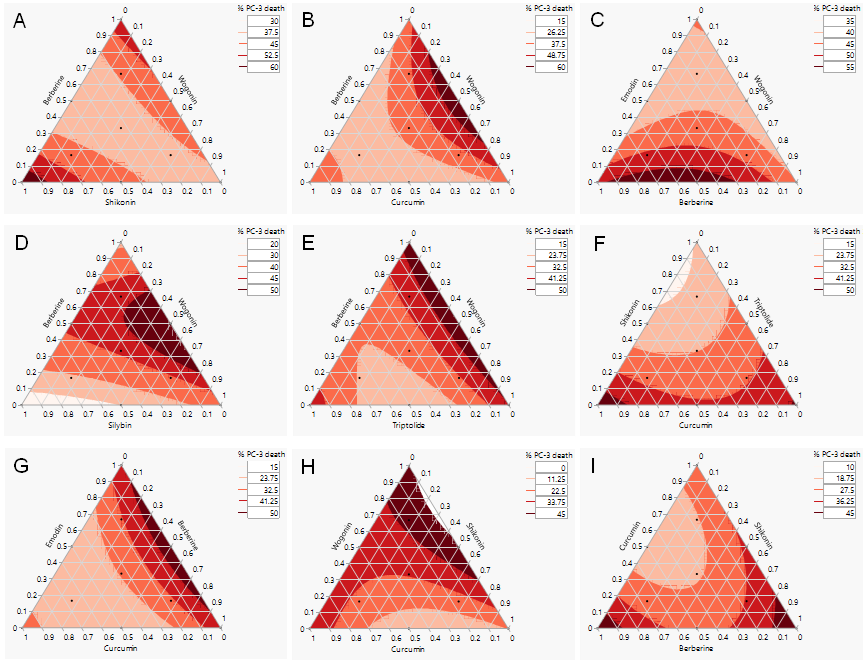


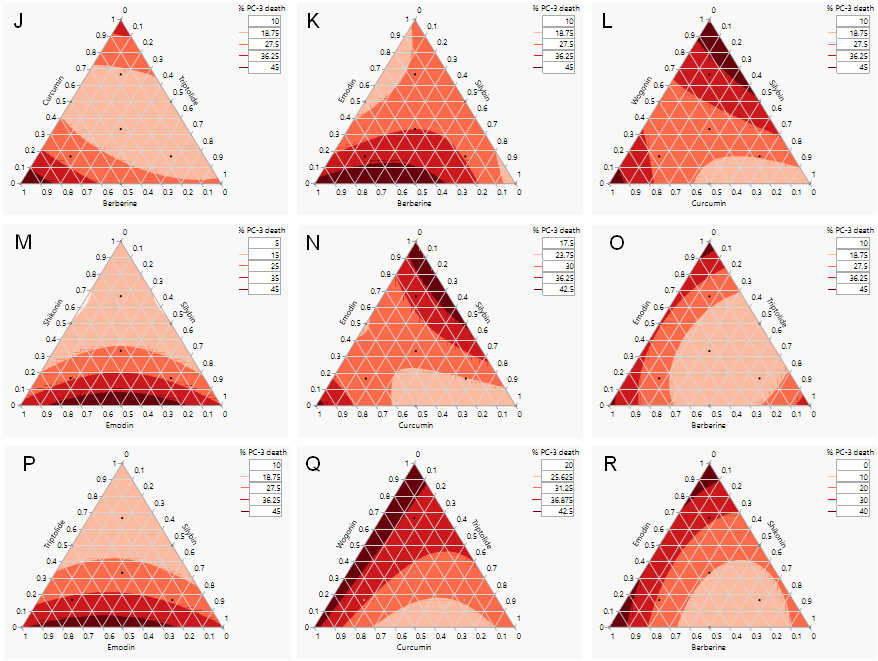


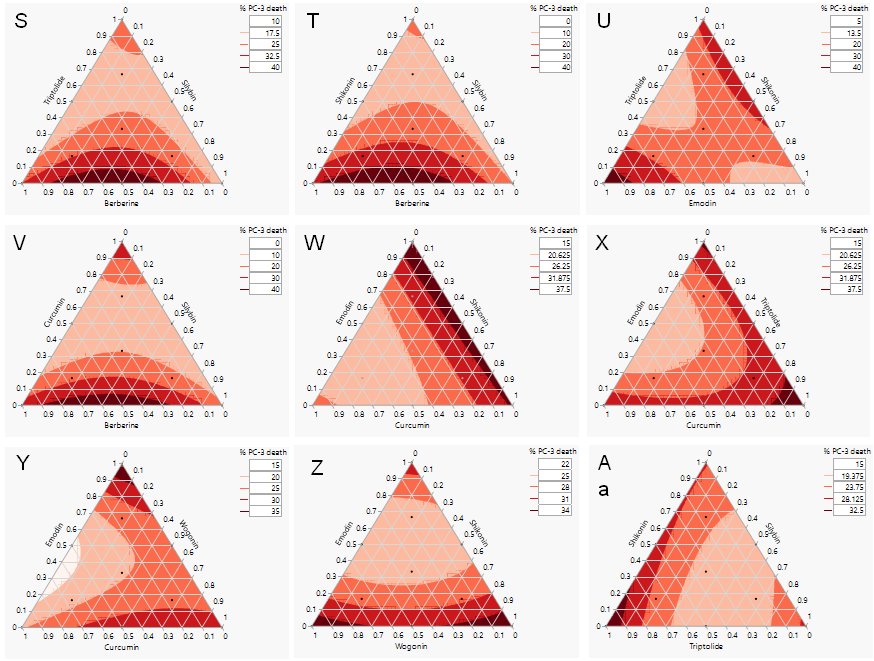


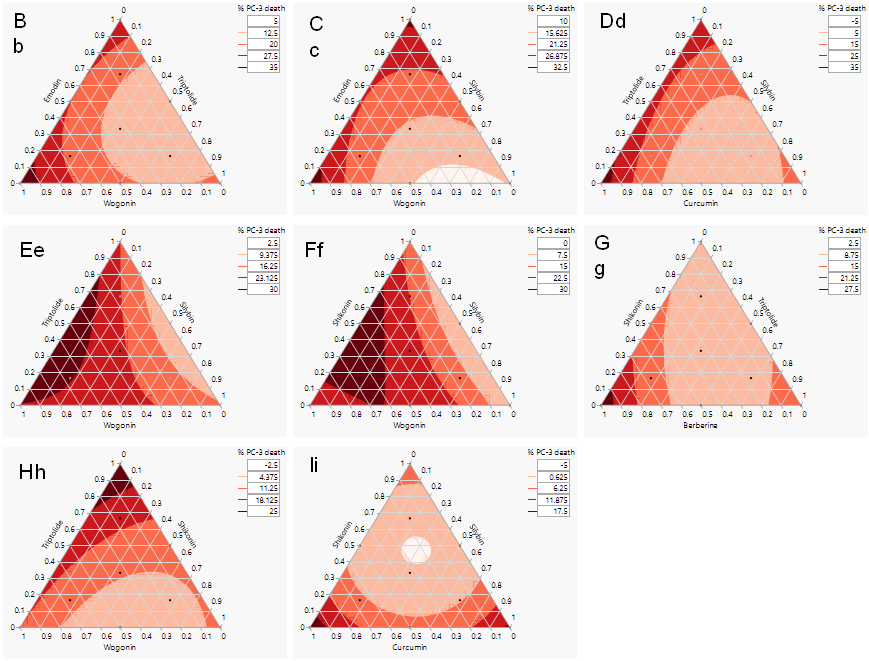


Supplemental Figure 3


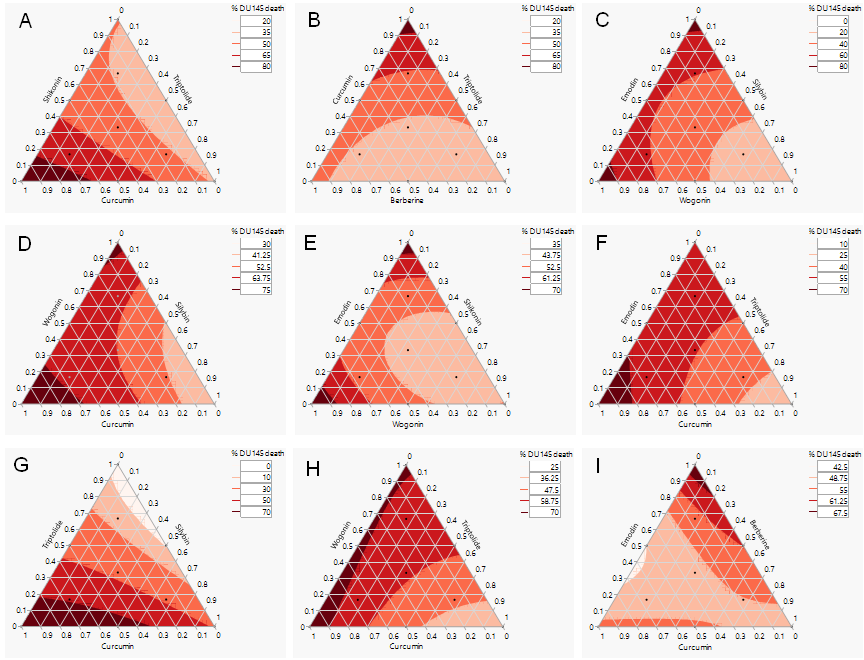


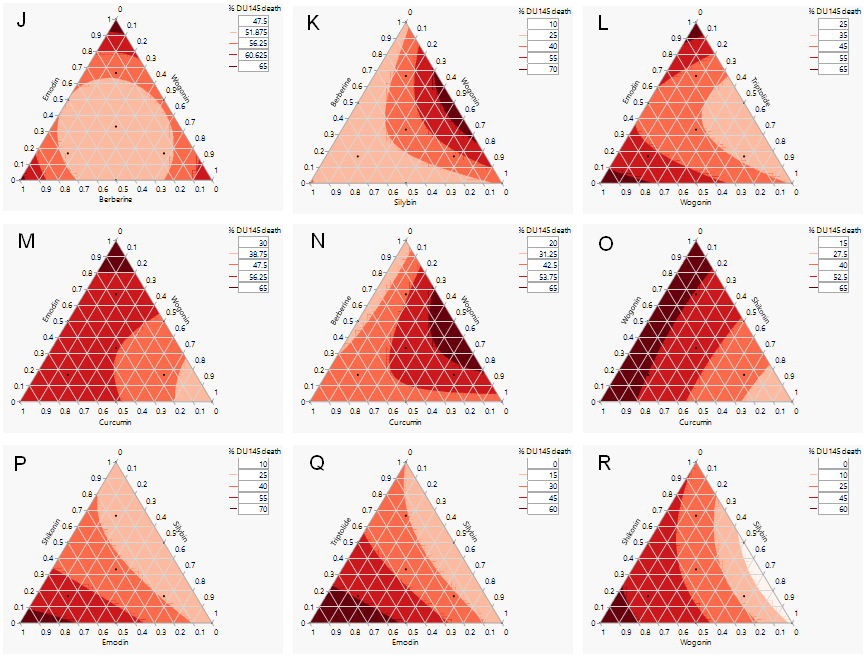


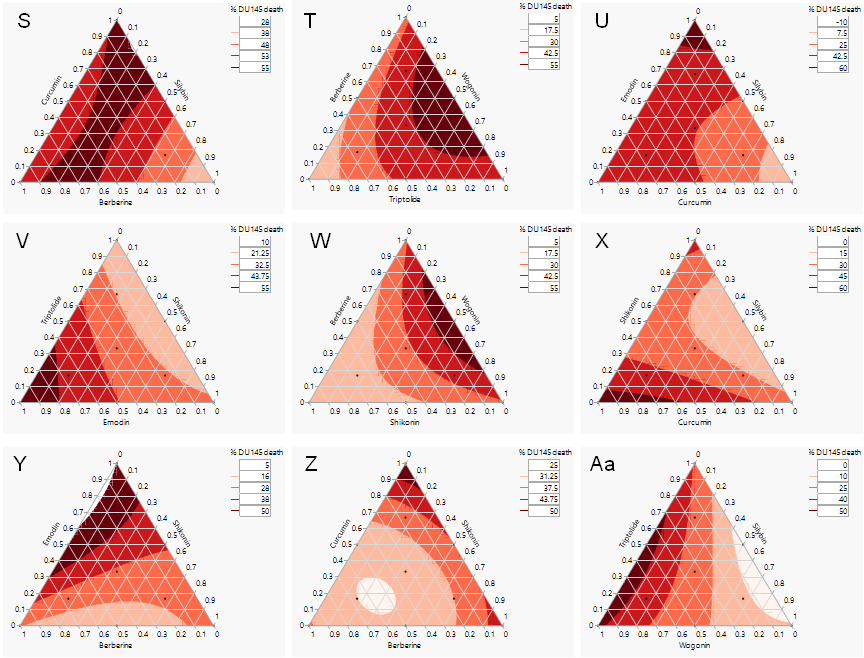


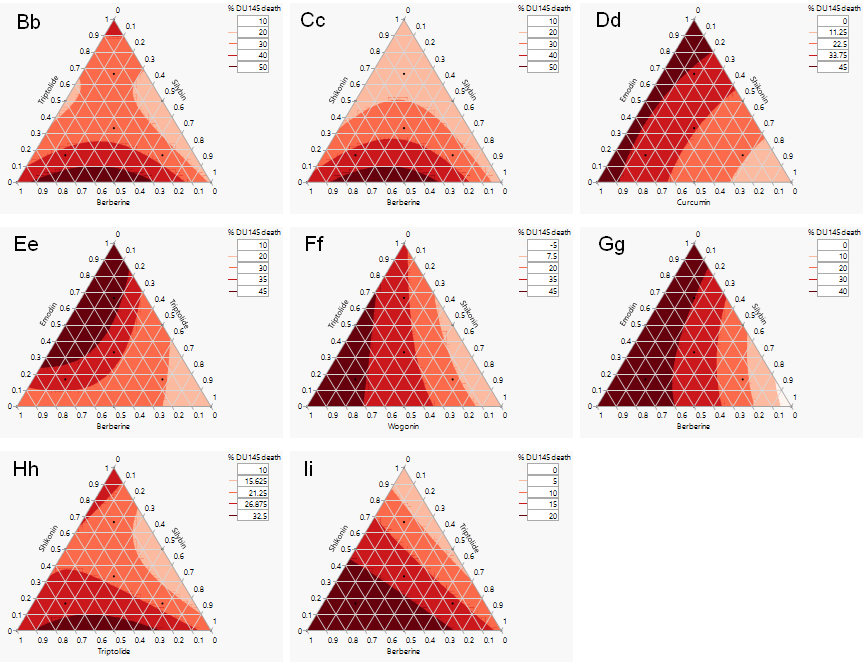


Supplemental Figure 4

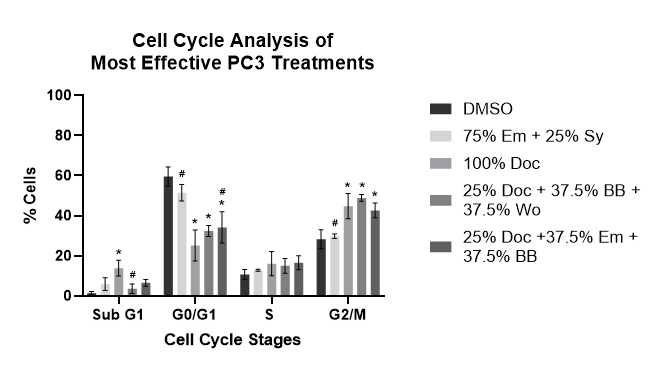
Supplemental Figure 5
